# Supplementary material for: Fast volumetric fluorescence lifetime imaging of multicellular systems using single-objective light-sheet microscopy
Source: Commun Biol. 2025 Nov 29;8:1785. doi: 10.1038/s42003-025-09221-2 (PMC12715196; doi:10.1038/s42003-025-09221-2)
Supplement: Supplementary file 3 — Description of Additional Supplementary files [file 42003_2025_9221_MOESM3_ESM.pdf]

Description of Additional Supplementary Files

File name: Supplementary Data 1

Description: Source data for graphs in figures 1-4

File name: Supplementary Movie 1

Description: 3D Multiplexing. The video shows soSPIM-FLIM imaging of an organoid in 3D, multiplexed via the lifetime. Membranes are stained with Flipper-TR and nuclei are labelled with GFP. Firstly, intensity images in a few z-planes are shown. In one zplane, a FLIM-image and the subsequent multiplexed image is shown (see methods). We then show the multiplexed images across the entire depth range. Acquisition time is 1.1 s per 2D plane and the scale bar is 25 µm.

File name: Supplementary Movie 2

Description: –3D Time-lapse Tension Imaging. The video shows 3D time-lapse FLIM of a live organoid stained with Flipper-TR, first showing 2d Page 8 of 17 planes in increasing depth and then the change of one plane over time. The lifetime scale corresponds to the long lifetime component of a two-component decay with the short lifetime fixed (see methods). Scale bar is 25 µm.
